# Rapid detection of internalizing diagnosis in young children enabled by wearable sensors and machine learning

**DOI:** 10.1371/journal.pone.0210267

**Published:** 2019-01-16

**Authors:** Ryan S. McGinnis, Ellen W. McGinnis, Jessica Hruschak, Nestor L. Lopez-Duran, Kate Fitzgerald, Katherine L. Rosenblum, Maria Muzik

**Affiliations:** 1 Department of Electrical and Biomedical Engineering, University of Vermont, Burlington, VT, United States of America; 2 Department of Psychiatry, University of Vermont, Burlington, VT, United States of America; 3 Department of Psychology, University of Michigan, Ann Arbor, MI, United States of America; 4 Department of Psychiatry, University of Michigan, Ann Arbor, MI, United States of America; Department of Psychiatry and Neuropsychology, Maastricht University Medical Center, NETHERLANDS

## Abstract

There is a critical need for fast, inexpensive, objective, and accurate screening tools for childhood psychopathology. Perhaps most compelling is in the case of internalizing disorders, like anxiety and depression, where unobservable symptoms cause children to go unassessed–suffering in silence because they never exhibiting the disruptive behaviors that would lead to a referral for diagnostic assessment. If left untreated these disorders are associated with long-term negative outcomes including substance abuse and increased risk for suicide. This paper presents a new approach for identifying children with internalizing disorders using an instrumented 90-second mood induction task. Participant motion during the task is monitored using a commercially available wearable sensor. We show that machine learning can be used to differentiate children with an internalizing diagnosis from controls with 81% accuracy (67% sensitivity, 88% specificity). We provide a detailed description of the modeling methodology used to arrive at these results and explore further the predictive ability of each temporal phase of the mood induction task. Kinematical measures most discriminative of internalizing diagnosis are analyzed in detail, showing affected children exhibit significantly more avoidance of ambiguous threat. Performance of the proposed approach is compared to clinical thresholds on parent-reported child symptoms which differentiate children with an internalizing diagnosis from controls with slightly lower accuracy (.68-.75 vs. .81), slightly higher specificity (.88–1.00 vs. .88), and lower sensitivity (.00-.42 vs. .67) than the proposed, instrumented method. These results point toward the future use of this approach for screening children for internalizing disorders so that interventions can be deployed when they have the highest chance for long-term success.

## Introduction

Nearly 1 out of every 5 children experience an internalizing disorder (19.6% anxiety, 2.1% depression) during childhood [1,2]. Anxiety and depression (collectively internalizing disorders) are chronic conditions that start as early as the preschool years [3,4] and impair a child’s relationships, development, and functioning [5–9]. If left untreated, childhood internalizing disorders predict later health problems including substance abuse [10,11], development of comorbid psychopathology [12–14], increased risk for suicide [15], and substantial functional impairment [16,17]. These negative long-term outcomes reveal the high individual and societal burden of internalizing disorders [18] and make clear the need for effective early interventions.

Thanks to greater neuroplasticity, interventions can be very effective in this population if disorders are identified early in development [19]. However, the current healthcare referral process usually involves parents reporting problem behaviors to their pediatrician and, if functionally impairing, the child is then referred to a child psychologist or psychiatrist for a diagnostic assessment. Children with internalizing disorders, where symptoms are inherently inward facing, are less likely than those with externalizing disorders to be identified by parents or teachers as needing professional assessment ([20]; for review see [21]), thus preventing or delaying their access to early intervention. Children under 6 have the highest rate of unmet needs [22]. For example, as little as 3% of 4 year-olds with a clinical diagnosis receive the necessary professional mental health intervention [23]. This points to the need for standardized screening tools for internalizing disorders in young children.

Even if referred, current diagnostic assessments have been shown to capture only the most severely impaired preschoolers, but miss a large number of children who may go on to develop additional clinical impairments [24,25]. Providers try to improve these assessments by considering multi-informant reports from children, parents, and teachers, but these also have limitations. For example, children under the age of eight are unreliable self-reporters [26–28], and parental report of child problems are often inaccurate [29–31] as the unobservable symptoms characteristic of internalizing disorders (e.g., thoughts and emotions), are difficult to identify and thus go underreported [31]. Parents who have an internalizing disorder themselves are known to over-report unobservable symptoms [32], increasing complexity of this problem. Thus, there is a clear and unmet need for objective markers of internalizing disorders that can be incorporated into new screening tools for all children.

Observational methods for assessing psychopathology ‘press’ for specific behaviors and affect [33] and have high research and clinical utility [34]. One example, known as a mood induction task, engages a child in a short laboratory-based activity meant to induce expected negative or positive emotions. To provide objective markers of psychopathology, researchers often utilize a behavioral coding technique on video recordings of the task, where at least two researchers watch the video recordings and assign scores based on child verbalizations or facial and body movements (e.g., see [35]). Behavioral coding has been shown to identify valid risk markers for childhood psychopathology using a variety of mood induction activities [36,37]. However, it has significant drawbacks that limit clinical utility, including the need for extensive training in a standardized coding manual and the hours required to watch and score video recordings of the task, while also consensus scoring a percentage of participants (often one out of five) to ensure reliability [38]. While it is clear that observational methods can provide objective markers of psychopathology, the complexity and resources required for behavioral coding prevent its use as a screening tool for childhood internalizing disorders.

New advances in wearable sensors present the opportunity to track child movement without the need for extensive training or time to watch and score task videos. Our previous work has described the use of a wearable inertial measurement unit (IMU), composed of a three-axis accelerometer and three-axis angular rate gyroscope, for tracking child motion during a standardized fear induction task [39–42]. Kinematical measures extracted from these data were associated with other known measures of risk for internalizing disorders and confirmed expected temporal characteristics of the task in a small (N = 18) sample of children [40]. In a larger sample (N = 62), IMU data, but not behavioral codes, were associated with parent-reported child symptoms and exhibited statistically significant differences between children with and without an internalizing diagnosis [41]. This *instrumented* mood induction task provides an objective measure of child motion without the limitations of behavioral coding and, when taken with these preliminary results, has the potential to be used as a screening tool for childhood internalizing disorders.

Advancing the use of this instrumented mood induction task as a tool for identifying children with an internalizing disorder requires establishing a model of the complex relationship between kinematical measures extracted from wearable sensor data and diagnosis. A data driven approach, like machine learning, is ideally suited for this task, and has been leveraged for this use in a variety of conditions including, for example, Multiple Sclerosis [43–45], Parkinson’s Disease [46,47], and Atrial Fibrillation [48]. In this case, the wearable sensor time series captured from each child during the mood induction task provides a complete, albeit high dimensional (i.e., an IMU sampling at 100 Hz for a 20-second task yields 12,000 data points), picture of their motion. However, by computing a smaller number of *features* (e.g., mean, kurtosis) that each explain a different pattern inherent to the data, the dimensionality of the high-dimensional time series can be reduced. This process of defining the set of features that is able to capture important aspects of the raw data is known as *feature engineering*. The process of *machine learning* is in then training a statistical model to recognize the relationship between these objective measures of a child’s motion during the task and their diagnosis. This approach allows for the realization of much more complex relationships then would be possible from theory-based modeling alone. These efforts form one facet of the burgeoning field of digital medicine [49,50], but notably the use of these techniques for improving childhood mental health is just beginning, with efforts focusing primarily on improving access to care through mobile delivery methods [51]. Thus, the use of machine learning and wearable sensors for advancing the state of childhood mental health screening represents a novel contribution to the field of digital medicine.

To further investigate the potential of this instrumented mood induction task as a screening tool for childhood psychopathology, we explore the use of machine learning to develop statistical models for identifying children who have an internalizing disorder. Specifically, this paper builds upon two of our recent conference papers [39,42] by presenting a detailed description of the modeling methodology and performance, an analysis of kinematical measures most discriminative of internalizing diagnostic status, and a comparison to the performance of models trained on parent-reported child symptoms.

## Method

### Participants

Studies had approval from the University of Michigan Institutional Review Board (HUM00091788; HUM00033838). Participants included 63 children (57% female) and their primary caregivers (95.2% mothers). Participants were recruited from either an ongoing observational study (Bonding Between Mothers and Children, PI: Maria Muzik; *n* = 14) or from flyers posted in the community (*n* = 14) and psychiatry clinics (*n* = 35) to obtain a sample with a wide range of symptom presentations. Eligible participants were children between the ages of 3 and 8 who spoke fluent English and whose caregivers were 18 years and older. Exclusion criteria were suspected or diagnosed developmental disorder (e.g., autism), having a serious medical condition, or taking medications that affect the central nervous system. The resulting sample of children were aged between 3 and 7 years (M = 5.25 SD = 1.10), was 65% White non-Latinx, and 82.5% lived in two-parent households. Twenty participants (32%) had an annual household income of greater than $100,000.

Multimodal assessments, including diagnostic interviews, were conducted for 62 of the children between August 2014 and August 2015. Based on these multimodal assessments and consensus coding, 21 participants were identified as having an internalizing diagnosis (current (n = 17), past (n = 4)) according to DSM-IV (Diagnostic and Statistical Manual of Mental Disorders, 4th. Edition). Diagnostic details are provided in Table 1.

**Table 1:** Diagnostic characteristics of the sample.

Primary Diagnoses	n = 22	Secondary Diagnoses	n = 10
Post-traumatic Stress Disorder	5	Specific Phobia	2
Anxiety/Depression Not Otherwise Specified	5	Social Anxiety Disorder	2
Adjustment Disorder	4	Depression	1
Separation Anxiety Disorder	4	Anxiety Not Otherwise Specified	1
Specific Phobia	3	Attention Deficit Hyperactive Disorder	2
Depression	1	Oppositional Defiant Disorder	2
Attention Deficit Hyperactive Disorder	1		

Number of subjects with specific primary and secondary diagnoses. Note that this includes both internalizing and externalizing diagnoses.

### Procedure

Child and caregiver were brought into the university-based laboratory and provided written consent to complete a battery of tasks. Caregivers completed self- and parent-report questionnaires and a diagnostic interview to assess for child psychiatric diagnoses while children underwent a series of behavioral tasks in an adjacent room. Behavioral tasks were designed to elicit fear responses and positive affect. Participants were compensated for their time.

Herein, we consider a subset of data from the larger study by examining participant response to a single behavioral task designed to elicit fear (the ‘Snake Task’), as well as the diagnostic interview and questionnaires used to assess internalizing symptoms and diagnoses. The Snake Task has been shown to induce anxiety and fear in young children [52,53]. This task is standardized and all research assistants were trained to carry out the task according to protocol. The total task duration was approximately 90 seconds, and task behaviors were conceptually segmented into three temporal phases [54]: 1) Potential Threat: The child was led into a novel, dimly lit room, unsure of what was inside while the administrator gave scripted statements to build anticipation such as “I have something in here to show you” and “Let’s be quiet so it doesn’t wake up”. The administrator led the child slowly toward the back of the room where a terrarium was covered with a blanket, gesturing for them to follow until they paused within 1 foot of the terrarium; 2) Startle: The child was startled by the administrator rapidly uncovering the terrarium and bringing the fake snake from inside to the child’s eye level several inches from their face; 3) Response Modulation: The child was encouraged to touch the snake if they wanted, to ensure it was fake, and was reassured verbally (e.g. “It’s just a silly toy snake”) as needed, remaining with the snake until the administrator gestured them to leave the room and end the task. Following the task, children transitioned to free play with the task administrator to regulate and debrief about their experience.

#### Questionnaires

The Child Behavior Checklist (CBCL) is a parent-completed questionnaire designed to assess child problem behaviors [55]. The scale consists of 120 items related to behavior problems across multiple domains. Items are scored on a three-point scale ranging from “not true” to “often true” of the child. Responses result in global T scores for externalizing, internalizing, and total problems, as well as a number of empirically based syndrome scales and disorder-based scales. Only scales available in both versions (ages 1.5–5 and 6–18) were used in subsequent analyses. The CBCL has well established validity and reliability (see [56]).

Subject demographic information was collected using a questionnaire that includes questions regarding child race, gender and family income.

#### Clinical interview

Trained clinical psychology doctoral students, or postdoctoral fellows, conducted a single structured clinical interview with each child’s caregiver. The current study used a version of The Schedule for Affective Disorders and Schizophrenia for School-Age Children Present and Lifetime Version (K-SADS-PL) modified for use with preschool-aged children [57]. In this diagnostic interview, the clinician spent up to two hours with the caregiver assessing symptoms of past and current child psychiatric disorders. Interviewers received monthly (or more frequent) supervision by a licensed psychologist and psychiatrist, wherein all cases were reviewed by all clinicians and the supervisor. Final diagnoses were derived via clinical consensus using the best-estimate procedures [58] to integrate a holistic picture based on child and parent report, family history, and other self-report symptom checklists. It is worth noting that team-based assessment and consensus diagnosis are most often only conducted in research contexts, the resulting diagnoses can be considered a true gold-standard, and this practice is not representative of the diagnostic procedures used in the majority of clinical contexts.

#### Wearable sensor signal processing and feature extraction

During the behavioral battery, child motion was tracked using a belt-worn IMU (3-Space Sensor, YEI Technology, Portsmouth, OH, USA) secured around the waist at approximately the location of the body center of mass. Acceleration and angular velocity data were sampled by the device at approximately 300 Hz, down-sampled to 100 Hz, and low-pass filtered using a fourth-order Butterworth IIR filter with a cutoff frequency of 20 Hz in software prior to use. These data were fused to determine device orientation as a function of time using the complementary filtering approach described in [40,59]. Device orientation was used to resolve raw IMU measurements of acceleration and angular velocity in a world-fixed reference frame that has one axis directed vertically upwards. These data were further decomposed into vertical acceleration and angular velocity (*av* and *ωv*, respectively), and the vector magnitude of horizontal acceleration and angular velocity (*ah* and *ωh*, respectively). Orientation estimates were also used to compute tilt (*α*) and yaw (*γ*) angles of the participant as a function of time yielding six time series (*ah*, *av*, *ωh*, *ωv*, *α*, *γ*) for further analysis. The interested reader may refer to [40], for a detailed description of this approach.

Time series were segmented into the three conceptual phases [54]: Potential Threat (20 seconds, from 23 to 3 seconds prior to the moment of startle), Startle (6 seconds, from 3 seconds prior to 3 seconds post the moment of startle), and Response Modulation (20 seconds, from 3 seconds to 23 seconds post the moment of startle) and signal features were extracted from each. Signal features included mean, root mean square (RMS), skew, kurtosis, range, maximum, minimum, standard deviation, peak to RMS amplitude, signal power within specific frequency bands (i.e., 0–0.5 Hz, 0.5–1.5 Hz, 1.5–5 Hz, 5–10 Hz, 10–15 Hz, 15–20 Hz, all frequencies greater than 20 Hz), and the location and height of peaks in the power spectrum and autocorrelation of the signal. This yielded a total of 29 features from each of the six time series, or 174 total features, from each phase of the task. Signal processing and feature extraction were performed in MATLAB (Mathworks, Natick, MA, USA), using source code available from [60].

#### Statistical models for identifying internalizing diagnosis

A supervised learning approach was used to create binary classification models that relate features from the IMU-derived signals to internalizing diagnosis derived from the K-SADS-PL and clinical consensus. Models were created using features from each temporal phase of the task. Performance of the classifiers was established using leave-one-subject-out (LOSO) cross validation. In this approach, features from 61 of the 62 subjects were partitioned into a training dataset and converted to z-scores prior to performing Davies-Bouldin Index [61] based feature selection to yield the 10 features with zero mean and unit variance that best discriminate between diagnostic groups. These features were used to train a logistic regression for predicting internalizing diagnosis. The same 10 features were extracted, converted to z-scores based on parameters (e.g. mean, variance) from the training set, and used as input to the model for predicting the diagnosis of the one remaining test subject. This process was repeated until the diagnosis of each subject had been predicted. Logistic regression was chosen herein to protect against overfitting given the relatively small (N = 62) sample, and because it requires minimal computational overhead for prediction enabling future deployment on resource-constrained devices.

We also examined the utility of the CBCL as a screening tool for internalizing diagnosis (according to the K-SADS-PL with clinical consensus) in this sample using previously-established clinical cutoffs (T score ≥ 70) for manualized use [55] and a more conservative cutoff (T score ≥ 55) suggested for improving screening efficiency [62].

Model performance was assessed in several ways. First, we examined classification performance by reporting accuracy, sensitivity and specificity with a score threshold for the logistic regression of 0.5. These metrics were computed following standard definitions [63]. Next, receiver operating characteristic (ROC) curves–which plot true positive rate against false positive rate for varying thresholds on the scores–were constructed for each classifier. Area under the ROC curve (AUC) was used to comment on the general discriminative ability of the classifiers [63]. Finally, a permutation test was conducted to examine these results in the context of results obtained by chance from this dataset [64]. To complete this test, we first approximated the distribution of possible error rates (error rate = number of incorrect predictions / total number of predictions = 1 –classification accuracy) for each classifier as a beta distribution parameterized by the number of incorrect predictions and the total number of observations, as indicated in [64,65], and randomly sampled 100 possible error rates from this distribution. Next, we repeated the model training process outlined previously for 100 random permutations of the diagnostic labels, computing the classification error rate for each. Finally, a paired-sample Mann-Whitney U-test was used to identify the temporal phases that yield classification models with error rates significantly different from those expected by chance from this dataset.

For models that reported significantly better error rates than those expected by chance, we further examined the features used as input to these models.

## Results

**Fig 1:**
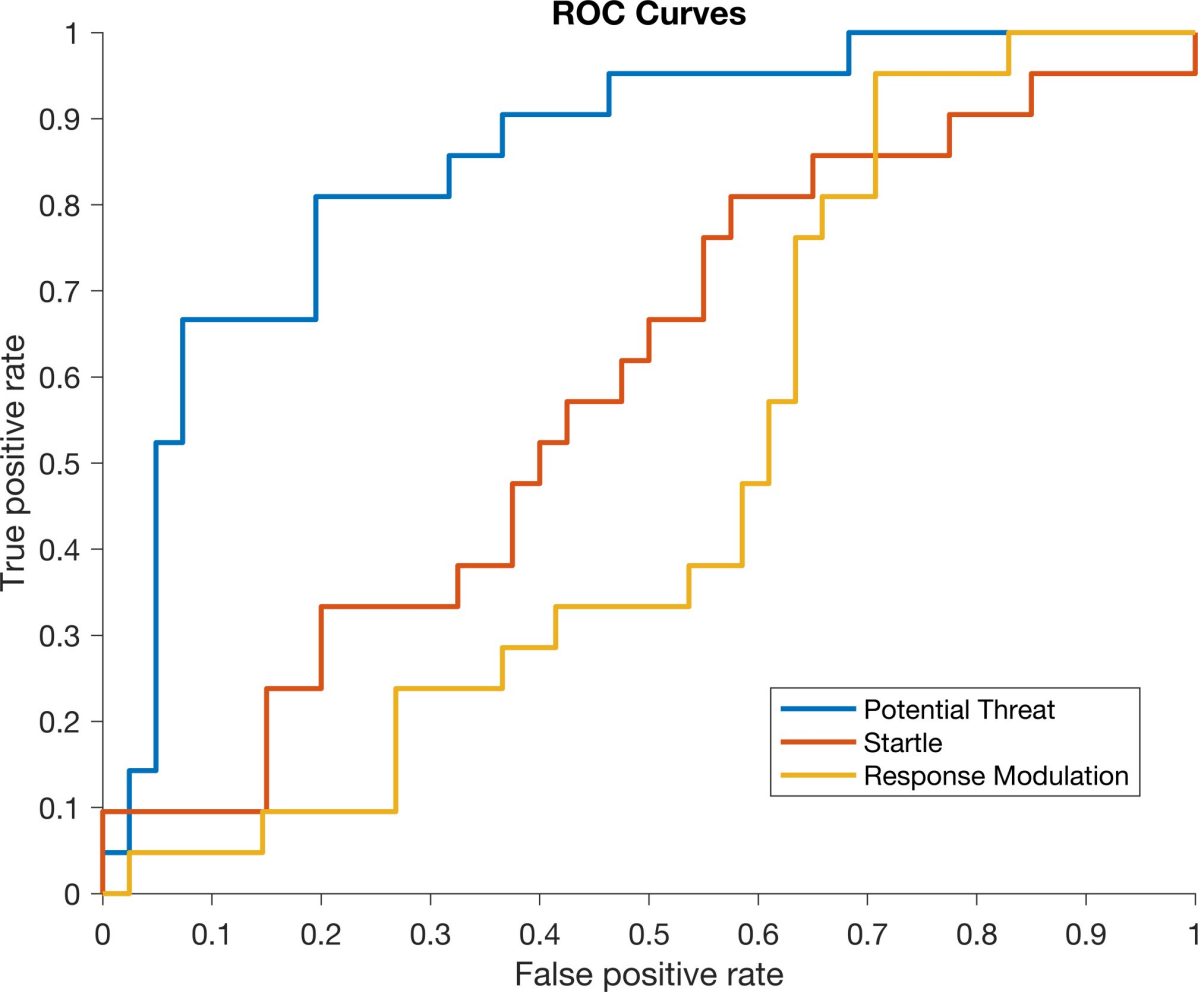
Receiver operating characteristic (ROC) curves for models trained to detect children with internalizing diagnoses. Curves for logistic regressions trained on data from the potential threat, startle, and response modulation phases of the snake task are indicated in blue, red, and yellow, respectively. The model trained on data from the potential threat phase performs better than the other models.

Performance of the logistic regression models trained for detecting children with internalizing diagnoses based on wearable sensor data from each temporal phase of the snake task are reported in Table 2. Metrics include accuracy, sensitivity, specificity, and area under the receiver operating characteristic curve (AUC). The model developed from data sample during the Potential Threat phase outperforms models from the Startle and Response Modulation phases (Accuracy: .81 vs. .58 and .52; Sensitivity: .67 vs. .33 and .29; Specificity: .88 vs. .71 and .63; AUC: .85 vs. .59 and .48). This performance difference is further revealed in the ROC curves of Fig 1, where models trained on data from the Potential Threat, Startle, and Response Modulation phases are shown in blue, red, and yellow, respectively. As indicated in the ROC curves, changing the score threshold for the logistic regression can alter its specific performance metrics. In this case, changing the threshold from .5 to .375 for the Potential Threat model maintains the overall accuracy (.81), decreases specificity (.88 to .81), and importantly increases sensitivity (.67 to .81). This could be important when considering use of this method for screening children for internalizing disorders.

**Table 2:** Performance characteristics of models developed for detecting children with internalizing diagnoses from wearable sensor data during each phase of the mood induction task.

	Accuracy	Sensitivity	Specificity	AUC
Potential Threat	.81	.67	.88	.85
Startle	.58	.33	.71	.59
Response Modulation	.52	.29	.63	.48

Accuracy, sensitivity, specificity and area under the receiver operating characteristic curve (AUC) for models trained using data from each temporal phase of the mood induction task (Potential Threat, Startle, Response Modulation).

**Fig 2:**
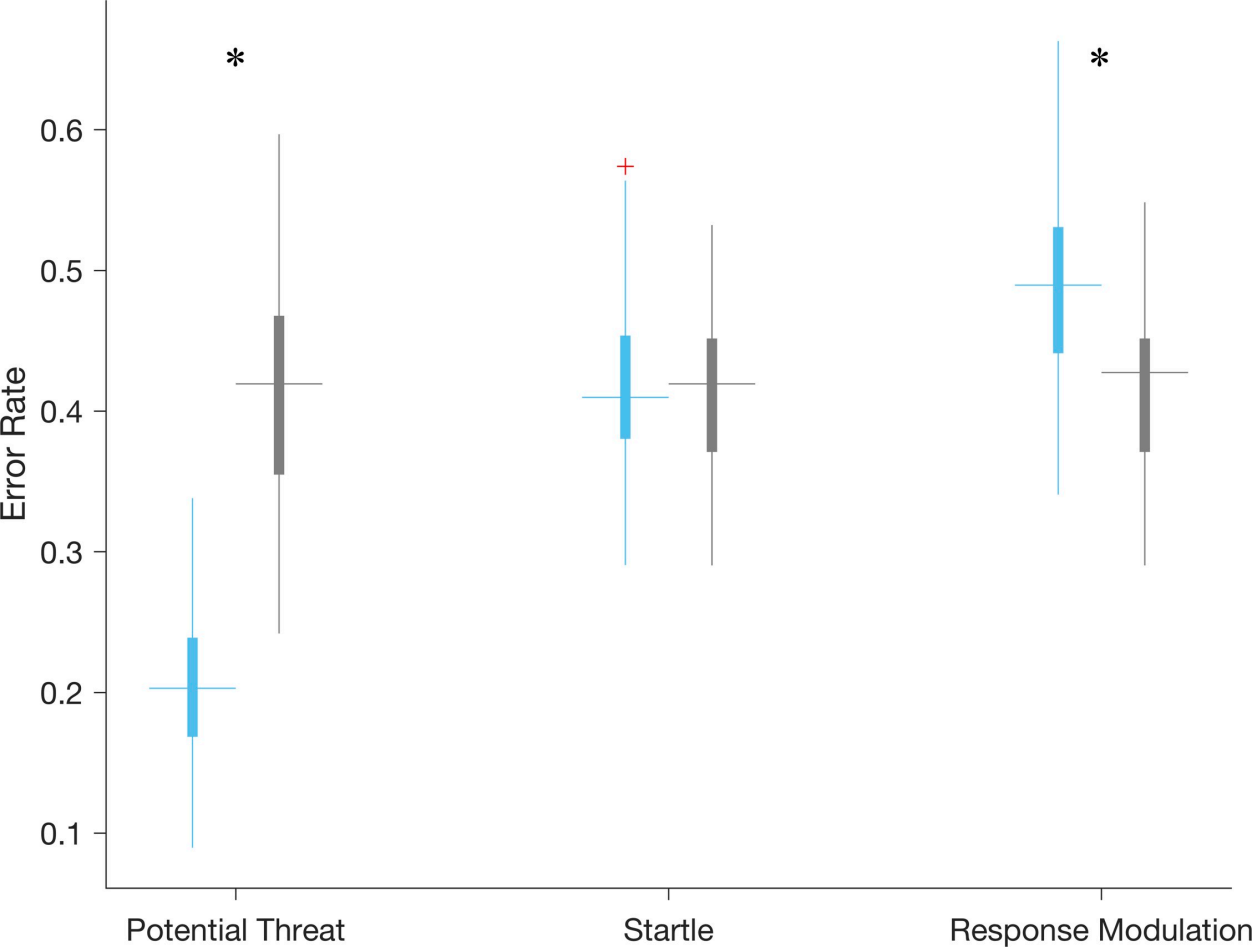
Boxplots of error rates for models trained to detect children with internalizing diagnoses compared to those due to chance for each temporal phase of the snake task. Error rates due to random chance determined via permutation test are shown in gray, while those from the actual data are in teal. Statistically significant differences are noted with an asterisk. The model trained on data from the potential threat phase is the only one to outperform random chance.

Results of the permutation test used for determining how the error rate of the classifiers trained on data from each phase compares to error rates expected by random chance from the dataset in are reported in the boxplot of Fig 2. Specifically, the distribution of error rates for the logistic regressions from each phase (teal) and the corresponding error rates achieved by chance (gray) are reported. Statistically significant differences between the median error rates achieved by the classifiers and by chance were observed in the Potential Threat (significantly lower error rate than expected by chance, p < .01) and Response Modulation (significantly higher error rate than expected by chance, p < .01) phases. Significant differences are indicated by asterisks in Fig 2.

**Fig 3:**
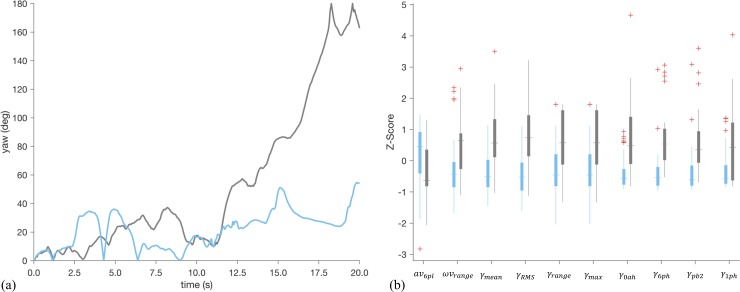
**Yaw angle time series from selected subjects (a) and boxplots of selected features from all subjects (b).** Time series data from a subject with an internalizing diagnosis is shown in gray, while that from a subject without is shown in teal. Similarly, gray boxplots correspond to data from subjects with a diagnosis while teal boxplots are from those without. The significant deviation in the yaw angle between subjects noted in (a) is reflected across all subjects in the boxplots of (b).

The results of Fig 2 indicate that the classifier developed from data sampled during the Potential Threat phase is the only model that provides a statistically significant improvement in classification error rate over random chance. To this end, we further examine the 10 features used as input to this model. Across the 62 iterations of the leave-one-subject-out cross validation, the following ten features were selected a minimum of 58 times: location of the 6^th^ peak in the power spectrum of *av* (*av*_6*pl*_), range of *ωv* (*ωv*_*range*_), mean of *γ* (*γ*_*mean*_), RMS of *γ* (*γ*_*RMS*_), range of *γ* (*γ*_*range*_), maximum of *γ* (*γ*_*max*_), the height of the peak at zero lag in the autocorrelation of *γ* (*γ*_0*ah*_), the heights of the 1^st^ and 6^th^ peaks in the power spectrum of *γ* (*γ*_1*ph*_) and *γ*_6*ph*_, respectively), and the signal power between 0.5–1.5 Hz in *γ* (*γ*_*pb*2_). Nine of the ten features in this list are directly related to the yaw angle (*γ*) of the subject (*ωv* is essentially yaw angular velocity), and we therefore report the *γ* time series from representative subjects with (gray) and without (teal) an internalizing diagnosis in Fig 3A. There is a significant divergence in the yaw angles achieved by each subject beginning roughly half-way through the phase that leads the subject with a diagnosis to end the phase facing in the opposite direction of where they started (*γ* ≅ 180°), which contrasts the subject without a diagnosis who ends the phase facing in roughly the same direction as when they started (*γ* < 60°). The differences noted in these representative subjects are consistent across the sample as evidenced by boxplots of the ten features used as input to the classifier presented in Fig 3B. Subjects with a diagnosis (gray) have higher values of *ωv*_*range*_, *γ*_*mean*_, *γ*_*RMS*_, *γ*_*range*_, and *γ*_*max*_, all of which confirm the divergence noted in Fig 3A is consistent across the sample.

Performance of the logistic regression models identifying internalizing diagnosis using elevated T scores (T score ≥ 70 and T score ≥ 55) for the internalizing broadband and two DSM-oriented scales (anxiety problems, depressive problems) of the CBCL are reported in Table 3. Metrics include accuracy, sensitivity, specificity, and AUC.

**Table 3:** Performance characteristics of models developed for detecting children with internalizing diagnoses from parent-reported child problems measured by the CBCL.

	Accuracy		Sensitivity		Specificity		AUC
Cutoff	70	55	70	55	70	55	70 & 55
Internalizing T Score	.68	.73	.00	.26	1.00	.95	.76
Anxiety Problems T Score	.75	.73	.21	.42	1.00	.88	.75
Depressive Problems T Score	.70	.72	.05	.26	1.00	.93	.79

Accuracy, sensitivity, specificity, and area under the ROC curve (AUC) for logistic regression models on parent-reported internalizing problems over the 6 months leading up to participation in the study as measured by the Child Behavior Checklist. Also included are two subscales of total internalizing problems (Anxiety Problems, Depressive Problems) oriented to DSM-IV criteria [56].

## Discussion

There is a significant need for a rapid and objective method for screening young children with internalizing disorders. We propose the use of data from a single wearable sensor during a 90-second fear induction task and machine learning to fulfill this need. Herein, we take an initial step toward this goal by training classifiers for detecting early indications of internalizing diagnoses using data sampled from each of three conceptually-segmented [54] temporal phases of a mood induction task, establishing their performance, and discussing the implications of these results. We further examine the specific features identified as being especially indicative of an internalizing diagnosis and discuss the behaviors described by these features in the context of internalizing disorders. The proposed approach is the first step toward creating an objective method for screening children for internalizing diagnoses rapidly and at low cost.

It is important to place these results in the context of previous work and existing diagnostic techniques. For example, these results compare favorably to our previous work, where k-nearest neighbor (k = 3) and logistic regression models were able to achieve 75% and 80% accuracy, respectively. The logistic regression employed herein achieves 81% accuracy, and we further report sensitivity (.67), specificity (.88), and ROC curves, quantities especially important when considering development of a tool used to screen for psychopathology. Of note, this logistic regression can be optimized for screening by adjusting the score threshold to yield an increase in sensitivity (.67 to .81) at the expense of a slight decrease in specificity (.88 to .81). Moreover, these results help to advance the use of wearable sensors and machine learning in childhood digital mental health, a burgeoning field that promises to improve access to, and speed of, mental healthcare. According to guides for classifying the accuracy of a diagnostic-screening test where AUC’s under .60 are considered fail, .60-.70 are poor, .70-.80 are moderate, .80-.90 are good, and .90–1 are excellent [66], IMU-derived features during the Potential Threat phase (AUC = .85) are considered good.

Investigation into specific temporal phases of the mood induction task demonstrated that it was the Potential Threat phase that differentiated between children with and without internalizing diagnoses as evidenced by the results of Table 2 and Fig 1. Children with internalizing disorders tended to turn away (*γ* ≅ 180°) from the ambiguous threat only when they were physically closest in the last 10 seconds of the phase (see Fig 3A). This may suggest that across depressive, anxiety and stress-related disorders, there is a shared anticipatory threat response which manifests physically in young children. Previous literature speculates that this type of response may be due to attention avoidance (attending away from the threat as shown in children with trauma and PTSD [67]) or emotional dysregulation (from attending to the threat in previous moments) [68].

Interestingly, the acute threat response during the Startle phase did not demonstrate clear differences by diagnostic status (e.g., see results of Figs 2 and 3 and Table 2). This could be due to heterogeneity of startle response across internalizing disorders, as previous research finds adults with moderate severity internalizing disorders (i.e., specific phobia) had heightened startles, healthy controls had low startle responses, and those with severe internalizing disorders (i.e., GAD, PTSD) had blunted startle responses [69]. A similar phenomenon may exist in our data, however larger sample sizes are needed to better assess this possibility. Alternatively, the significantly heightened avoidance motion (i.e., *γ*) during Potential Threat without a significantly heightened motion during Startle could suggest a physiological manifestation of “It wasn’t as bad as I thought it was going to be,” a cognitive shift often seen in anxious children after exposure therapy [70]. Regardless of other phases, ambiguous threat avoidance during potential threat contexts appears to unify internalizing disorders and differentiate them from controls (e.g., see results of Table 2 and Fig 3B).

We compare psychometric properties of the questionnaire-based parent-reported CBCL and IMU-derived feature models on child internalizing diagnosis as determined via K-SADS-PL with clinical consensus. CBCL-derived models for both cutoffs (55 and 70) exhibited slightly lower classification accuracy (.68-.75 vs. .81), slightly higher specificity (.88–1.00 vs. .88), lower sensitivity (.00-.42 vs. .67), and slightly lower AUCs (.75-.79 vs. .85) compared to IMU-derived models during the Potential Threat phase. CBCL subscale psychometrics in our study are similar to those from much larger studies (e.g., see [66,71]). Notably, both in our study and paralleled in these previous studies is the varied sensitivity of the CBCL, with some samples exhibiting sensitivities as low as .00-.38 [72] and some as high as .44 to .86 [73]. Overall, CBCL internalizing psychometrics across studies suggest room for improvement in internalizing screening efficiency, especially as it was consistently worse than that of externalizing screening efficiency [71]. The IMU-based results presented herein yield a minimum 60% improvement in sensitivity over that observed from the CBCL suggesting that this supplemental objective data may be especially helpful for increasing sensitivity.

Overall, this paper describes a methodology requiring very limited computational resources (e.g. compute 10 features from 20 seconds of wearable sensor data, use as input to a logistic regression) which points toward future deployment of this technique for identifying young children with internalizing disorders using resource-constrained but ubiquitous devices like mobile phones. This new approach reduces the time required for diagnostic screening while also establishing high sensitivity–which can help to reduce barriers and better alert families to the need for child mental health services. While these results can likely be improved and extended, and should be replicated, this is an important first step in connecting often overlooked children [20,21] to the help they need to both mitigate their current distress and prevent subsequent comorbid emotional disorders and additional negative sequelae [12,14,74].

### Limitations

This study is not without limitations. Future research should replicate and investigate our claims in a larger study with subjects at varying levels of risk for developing an internalizing disorder. Additionally, a larger sample size would allow examination of internalizing disorders without the presence of comorbid externalizing disorders, and also specific internalizing disorders to explore whether one disorder type yielded different motions than another. Future work may also explore alternative or additional device locations and more complex non-linear models for improving classification performance.

## Conclusion

The results presented herein demonstrate that, when paired with machine learning, 20 seconds of wearable sensor data extracted from a fear induction task can be used to identify young children with internalizing disorders with a high level of accuracy, sensitivity, and specificity. These results point toward the future use of this approach for screening children for internalizing disorders.

## Supporting information

S1 File IMU features, CBCL scales, and clinical diagnosis. (XLSX)
